# Tools to accelerate falciparum malaria elimination in Cambodia: a meeting report

**DOI:** 10.1186/s12936-020-03197-6

**Published:** 2020-04-15

**Authors:** Dysoley Lek, James J. Callery, Chea Nguon, Mark Debackere, Siv Sovannaroth, Rupam Tripura, Marius Wojnarski, Patrice Piola, Soy Ty Khean, Kylie Manion, Sokomar Nguon, Amber Kunkel, Lieven Vernaeve, Thomas J. Peto, Emily Dantzer, Chan Davoeung, William Etienne, Arjen M. Dondorp, Luciano Tuseo, Lorenz von Seidlein, Jean-Olivier Guintran

**Affiliations:** 1 Centre for Parasitology, Entomology and Malaria Control, Phnom Penh, Cambodia; 2 Mahidol-Oxford University Tropical Medicine Research Unit, Mahidol University, Bangkok, Thailand; 3 Malaria Consortium, Phnom Penh, Cambodia; 4 Centre for Tropical Medicine and Global Health, University of Oxford, Oxford, UK; 5 Armed Forces Research Institute of Medical Sciences, Bangkok, Thailand; 6 Institut Pasteur du Cambodge, Phnom Penh, Cambodia; 7 University Research Company Ltd., Phnom Penh, Cambodia; 8 London School of Hygiene and Tropical Medicine, London, UK; 9 Emerging Diseases Epidemiology Unit, Institut Pasteur, Paris, France; 10 University of California at San Francisco, San Francisco, USA; 11 Provincial Health Department, Battambang, Cambodia; 12 World Health Organization, Phnom Penh, Cambodia

**Keywords:** Cambodia, Case detection, Forest malaria, Malaria elimination, Mass drug administration, *Plasmodium falciparum*, Prophylaxis, Screening and treatment

## Abstract

Cambodia targets malaria elimination by 2025. Rapid elimination will depend on successfully identifying and clearing malaria foci linked to forests. Expanding and maintaining universal access to early diagnosis and effective treatment remains the key to malaria control and ultimately malaria elimination in the Greater Mekong Subregion (GMS) in the foreseeable future. Mass Drug Administration (MDA) holds some promise in the rapid reduction of *Plasmodium falciparum* infections, but requires considerable investment of resources and time to mobilize the target communities. Furthermore, the most practical drug regimen for MDA in the GMS—three rounds of DHA/piperaquine—has lost some of its efficacy. Mass screening and treatment benefits asymptomatic *P. falciparum* carriers by clearing chronic infections, but in its current form holds little promise for malaria elimination. Hopes that “highly sensitive” diagnostic tests would provide substantial advances in screen and treat programmes have been shown to be misplaced. To reduce the burden on *P. falciparum* and *Plasmodium vivax* infections in people working in forested areas novel approaches to the use of malaria prophylaxis in forest workers should be explored. During an October 2019 workshop in Phnom Penh researchers and policymakers reviewed evidence of acceptability, feasibility and effectiveness of interventions to target malaria foci and interrupt *P. falciparum* transmission and discussed operational requirements and conditions for programmatic implementation.

## Background

The last decade was marked by increased investments in falciparum malaria control associated with a decrease in malaria transmission in most endemic countries and specifically in the Greater Mekong Subregion (GMS) [1]. Early diagnosis and effective treatment of falciparum malaria is now widespread and more easily available. Even in the absence of additional interventions, it would be reasonable to assume that malaria, at least falciparum malaria, will slowly but steadily fade out in the coming years by maintaining access to early and effective case management combined with plans to increase the number of peripheral health care providers. The emergence and spread of *Plasmodium falciparum* strains resistant to the current first-line treatment, artemisinin-based combination therapy (ACT), presents a barrier to this approach in the GMS [2]. In the absence of an alternative readily available effective first-line treatment the “early diagnosis and effective treatment” strategy may fail and be followed by a resurgence in falciparum malaria transmission. It is, therefore, necessary to accelerate falciparum malaria elimination in the GMS as quickly as possible.

There is a consensus that reducing the vectorial capacity has an exponential impact on malaria transmission and hence vector control is critical in malaria elimination. Yet attempts to reduce exposure to competent vectors has been uniquely disappointing in Cambodia and the wider GMS. The reasons relate to the incredibly diverse vector population in the region, which in contrast to vectors in sub-Saharan Africa (SSA), are daytime, outdoor biters (exophilic/exophagic) [3, 4]. The most promising vector control strategies in SSA, long-lasting insecticide-treated bed nets and indoor residual insecticide spraying, are of limited use in Cambodia and the wider GMS. The few randomized controlled trials assessing the benefit of long-lasting insecticide-treated bed nets have been disappointing [5–7] and the same is true for indoor residual insecticide spraying [8–10]. The malaria vectors in the GMS bite mostly outdoors and the air-permeable materials (e.g. bamboo cladding) used in rural homes in the GMS are poorly suited for indoor residual insecticide spraying. Furthermore, the population at highest risk in SSA are young children sleeping in a home, usually in a brick or a wattle and daub house with their parents or guardians. The population at highest risk in Cambodia and most of the GMS are forest workers who sleep in mostly improvised shelters in forested areas where they work, eat, and socialize outdoors—activities that do not lend themselves to conventional vector control approaches [11, 12]. Militaries, another high-risk population working in the forest, face the similar challenges, being left unprotected during peak biting hours. Well-intended attempts to provide forest workers with long-lasting insecticide-treated hammock nets and insect-repellents have yet to show effectiveness in randomized controlled trials. Attempts to introduce spatial repellents for vector control have also shown limited promise [13]. The potential benefits of clothes and uniforms treated with insect-repellents or insecticides are currently under evaluation. Alternative approaches to outdoor vector control include larviciding, outdoor fogging and attractive toxic sugar baits [14–16]. Convincing policymakers to invest into these vector control approaches will require empirical evidence of effectiveness. Vector control is of critical importance in the elimination of malaria, yet the vector control community has not demonstrated that strategies which are successful in SSA can be successfully transferred to the GMS. Considering the local diversity of vectors, there is no easy, quick vector control solution on the horizon.

All countries in the GMS (Cambodia, Laos, Myanmar, Thailand, and Vietnam) have signed up to eliminate falciparum malaria before 2030, which requires efforts from political and public health stakeholders beyond the business-as-usual model [17]. There is a consensus that more work is needed in malaria elimination globally and specifically in the GMS, where drug resistance is now limiting the treatment options. What is far from clear is how the current malaria elimination efforts can be accelerated and which form of acceleration makes the largest, sustained impact. The Cambodia National Malaria Programne (CNMCP) has a detailed operational manual on falciparum elimination [18] adapted from generic global World Health Organization (WHO) guidelines [19, 20], but more precise guidance for foci management is needed. Over the last decade researchers in Cambodia have implemented enhanced surveillance methods to understand transmission patterns and improve passive case detection. The impact of a range of interventions, including mass drug administrations for falciparum malaria and mass screening and treatment projects, have been evaluated. To review the available evidence on active measures, a meeting was convened in Phnom Penh on 25 October 2019 entitled *“Interventions to locate malaria foci and interrupt transmission of Plasmodium falciparum. Review of evidence from recent and ongoing studies or operations*”. This report summarizes the presentations and conclusions from the meeting. Study findings presented during the meeting are cross-referenced and supplemented with published papers.

## Early diagnosis and effective treatment

**Fig. 1:**
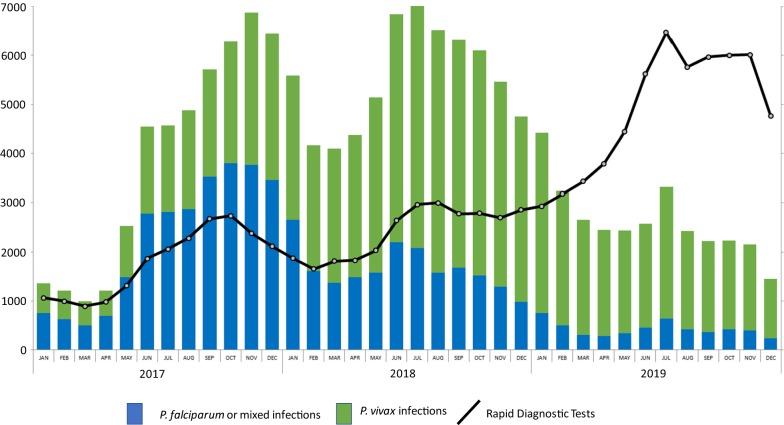
Monthly malaria cases reported by CNMCP information system—2017–2019

The foundation of the current progress in malaria control is access to early diagnosis and treatment. There is a broad consensus that increasing this access has the highest priority. Recent evidence for the success of this strategy comes from the roll out of malaria posts in Karen State, Myanmar which played an important part in the dramatic reduction of malaria transmission [21]. In 2009, Cambodia successfully introduced village malaria worker (VMW) programmes which have now been expanded country wide [22–26]. The main challenges in the ongoing case management of malaria are the continued provision and support of competent health care providers e.g. VMW, and the assurance that there are no stock outs of effective first-line malaria treatments. This basic provision of health care services has been challenging in Cambodia. The interruption of funding from the Global Fund of VMW programme during 22 months in 2016–2017 was followed by a large increase in reported malaria cases. The CNMCP reacted by launching an “intensified response plan” funded by reprogrammed Global Fund resources from the Regional Artemisinin-resistance Initiative (RAI) 2E grant [27]. The targeted response was restricted to the catchment areas of 45 health centres with the highest incidence of falciparum cases from 10 operational districts within the 7 provinces with remaining forested areas. The core strategy aims to facilitate access to “test-and-treat services” for forest goers by deploying about 200 Mobile Malaria Workers (MMWs) on the main routes in and out of the forest to supplement the already deployed VMWs based in villages. In addition to the change of first-line treatment in 2017, faster treatment provision is likely to have contributed to the drop in reported *P. falciparum* incidence between 2018 and 2019 (Fig. 1).

## How to locate high-risk populations and malaria foci?

In several areas of Cambodia, provincial malaria officers supported by WHO consultants are analysing granular spatial surveillance data and are mapping hotspots. In addition, several research groups are currently working on mapping transmission foci [28–32]. The major challenge remains to decide what effective actions can be implemented based on these surveillance data.

There is evidence that In Cambodia malaria transmission is mainly occurring in forests [11]. To improve the limited access forest goers have to early diagnosis and treatment, the Malaria Consortium piloted the deployment of mobile malaria posts near the Lao and Vietnam borders. The location of these posts needs to be regularly adjusted to account for forest goer movements. Considering malaria is mainly transmitted inside forests, an alternative approach could be to directly implement malaria elimination activities inside forests. All individuals found inside forests belong, by definition, to the highest risk population. Institut Pasteur of Cambodia (IPC) presented an on-going study with real-time GIS tracking of forest goers by trained operators who also complete questionnaires and collect blood spots for PCR from the forest goers. Preliminary unpublished data demonstrate the feasibility of tracking forest goers (FG) and second, a high proportion of forest goers are asymptomatically infected. A monthly Intermittent Presumptive Treatment of forest goers (IPTfg) prescribed by trained FGs based inside forests could have a dramatic impact on both falciparum and vivax malaria at the source of transmission.

## Reactive case detection

Following the implementation of early diagnosis and treatment programmes the next strategic step towards elimination are case investigations. The 1-3-7 strategy works well in China (Box 1). Subtle, but critical, differences in malaria epidemiology between Cambodia and China made it essential to adapt this strategy for Cambodia leading to the following components (1) Localize—case/foci surveillance, (2) Catch—intensification out of the forest and (3) Clear—focal interventions, including a reactive component. Each falciparum malaria case investigation starts with interviews in the village of the index case within 3 days of notification to determine whether the infection was acquired locally or outside the village. The interviews explore potential exposure based around the question—“*Did you sleep every night in this village within the last 2* *weeks?*”. The second phase consists of a focus investigation conducted by the operational district (OD) malaria staff within 14 days of detection. The investigation takes 4 days and includes a desk review, village mapping, household surveys and night capture of mosquitoes. Foci are classified according to receptivity (R1—High, R0—Low) and vulnerability (V1—High, V0—low) and the summary score determines the set of interventions to be undertaken.

As presented during the meeting, between 2013 and 2018 four research groups have been working on reactive case detection in four districts of Cambodia. Malaria Consortium (MC) in Pailin, University Research Company (URC) in Sampov Loun, Médecins Sans Frontières (MSF) in Preah Vihear, and the London School of Hygiene and Tropical Medicine (LSHTM) in Oddar Meanchey Province (Table 1). The case investigations yielded very few positive RDT results. With detection rates below 2% and a low diagnostic sensitivity in asymptomatic patients, it will be difficult to convince policymakers to continue supporting such programmes. More importantly, there is already some evidence to suggest that staff and the target communities experience fatigue and participation rates can be expected to dwindle.

**Table 1:** Effectiveness of re-active case detection in Cambodia

Location	Year	Organization	Index cases	Screened	Positive RDT	Positive PCR
Pailin	2013	MC	270	1898	9 (0.5%)	17 (0.9%)
Sampov Loun	2015–2018	URC	639	1946	15 (0.8%)	Not done
Preah Vihear	2016–2018	MSF	60	226	2 (0.9%)	8 (3.5%)
Oddar Meanchey^a^	2017–2018	LSHTM/HSD	192	1574	26 (1.6%)	66 (4.2%)

*MC* Malaria Consortium, *URC* University Research Company, *MSF* Médecins Sans Frontières, *LSHTM/HSD* London School of Hygiene and Tropical Medicine/Health & Social Development

^a^PACES trial–Pro-active case detection and community participation for the elimination of malaria study [68]

Box 1: The 1-3-7 strategy designed to guide and monitor malaria surveillance and response in China [33]

- 1: Case reporting within 1 day. Any confirmed and suspected malaria cases must be reported by law to the web-based government health information system within 24 h of diagnosis by the local health-care provider.
- 3: Case investigation within 3 days. Determine where the case originated (local or imported). All malaria cases should be confirmed and visited by the county-level China Centres for Disease Control, where the case is reported within 3 days.
- 7: Focus investigation and action within 7 days. The focus investigation should be conducted as soon as possible. If local transmission is possible or confirmed, targeted action to detect other infections and to reduce the chance of onward transmission is completed within 7 days by the county-level China CDC of the county where the patient resides and/or works. This last part includes the screening and treating of up 200 neighbours of the index case.

## Mass and focal screening and treatment

The mass screen and treat strategy is easier to accept by target populations than mass drug administration (MDA, described below), but there are questions regarding the impact of this strategy. Early experience in screening and treatment comes from a project conducted in 2010 in 20 villages in Pailin province by the CNMCP, supported by the WHO using a novel mobile PCR laboratory. Among the 6931 individuals screened, the prevalence of *P. falciparum* was less than 1% and 96% of the PCR-positive participants were asymptomatic [34]. This pilot intervention in the context of the containment plan was found to be too demanding and was abandoned. Since then, research groups have tested pro-active case detection with Rapid Diagnostic Tests (RDTs) in Cambodian villages between 2017 and 2019. URC conducted studies in Pursat province, MSF in Preah Vihear [35] and the LSHTM in Oddar Meanchey Province [36]. Researchers from the University of San Francisco (UCSF) presented a similar study conducted in southern Lao [37]. The tested intervention was either Mass Screening and Treatment (MSAT) with the whole population targeted or Focal Screening and Treatment (FSAT) restricted to highest risk groups, usually forest goers or people with fever. The findings presented during the meeting suggest that the prevalence of infection is extremely low even in the most at-risk villages (Table 2). There was no discernible additional benefit using “highly sensitive” rapid diagnostic tests (HSRDTs). The cost effectiveness of such expensive and logistically demanding operations is questionable since there is no pragmatic evidence or convincing mathematical models to suggest a test and treat strategy could result in the interruption of the transmission.

**Table 2:** Effectiveness of pro-active case detection for detection of *P. falciparum* in Cambodia

Location	Year	Organisation	Type	Screened	Positive		
					RDT	HSRDT	PCR
Pailin	2010	CNMCP	MSAT	6931	Not done	Not done	60 (0.9%)
Preah Vihear	2017–2019	MSF	MSAT	11,902	Not done	137 (1.2%)	8 (3.5%)
			FSAT	8670	44 (0.5%)	Not done	Not done
Oddar Meanchey	2017–2018	LSHTM/HSD	MSAT	2051	10 (0.5%)	10 (0.5%)	10 (0.5%)
Pursat	2019	URC	FSAT	641	1 (0.2%)	Not done	Not done

*MSAT* mass screening and treatment, *FSAT* focal screening and treatment; *CNMCP* Cambodian National Malaria Control Programme, *MSF* Médecins Sans Frontières, *LSHTM/HSD* London School of Hygiene and Tropical Medicine/Health & Social Development, *URC* University Research Company

Researchers from IPC simulated the impact of MDA and the screen and treat approach (Additional file 1: Figure S1). Under the MDA and the screen-and-treat strategies, the number of infections treated was simulated according to five scenarios, from coverage of the entire community to subpopulations within the community. Assuming that conventional RDTs can detect 30% of infections detected by uPCR, screening will miss at least 70% of the *P. falciparum* infections in the community. More realistically not all invited community members will participate in the mass screening and not all test-positives will clear their infections and far more than 70% of *P. falciparum* infected community members will remain untested and potentially transmit malaria. Focal screening of high-risk sub-populations is more cost-effective but will leave the infected community members who are not part of the targeted subpopulation unscreened and therefore untreated. The treatment of around 30% of infected community members in a best-case scenario, when the entire community is screened, will be substantially below any reasonable threshold for malaria elimination and is, therefore, unlikely to have an impact on transmission. The simulations suggest that mass drug administration (MDA) to entire communities or sub-populations at highest risk would be, at least in theory, more effective than a screen and treat approach (the challenges of MDA are discussed below).

It has been shown repeatedly when compared to presumptive treatment, communities prefer to be tested and only be treated if infected over [34, 38, 39]. Mass screening and the exclusive treatment of infected participants will benefit the infected individual, but it can only reduce transmission substantially if most infected people are correctly identified. And here is the crux of the screen and treat approach in malaria: the current tests are not sensitive enough to be up to the task. The most sensitive diagnostic test for *P. falciparum* infections is currently ultrasensitive quantitative polymerase chain reaction (uPCR) based on high volume blood samples. The processing of samples for uPCR takes days and is costly (laboratory costs alone are ~ 40 USD per sample analysed). More importantly, even this state-of-the art diagnostic approach will only detect 70% of individuals with *P. falciparum* in peripheral blood samples [40]. There are no credible estimates of how many infected people only carry sequestered parasites with little or no parasites in the peripheral circulation. A new generation of highly sensitive RDTs (HS-RDTs) has been recently developed [36, 41]. Investigators presented findings from two recent studies conducted in the GMS suggesting that the gains of HS-RDTs compared to conventional RDTs are minimal (< 10% increase in sensitivity) and will not increase detection rates substantially (Box 2). A successful screen and treat strategy for malaria elimination would require an ideal diagnostic test that is non-invasive, detects even the lowest parasite densities, provides results immediately and all this at an affordable price in field settings. Specificity and the problem of over-treatment (treatment based on false positive test results) is a minor issue in a screen and treat strategy, considering the comparatively widespread over-treatment in MDA campaigns.

Box 2: The sensitivity of screening tests **LSHTM** conducted the PACES (Proactive case detection and community participation for the elimination of malaria study.) trial in Oddar Meanchey Province, Cambodia (14 Health facilities, 130 villages with VMWs; 20 months).

- LAMP vs. qPCR
- Sensitivity 51.6%, Specificity 96.8%
- By subgroup (forest goers)—Sens 73.8%, Spec 83.8%
- Highly Sensitive-RDT (HS-RDT) and conventional RDT vs. qPCR
- HS-RDT vs. RDT shows similar results during active case detection
- HS-RDT—Sens 53.8%, Spec 98.2%
- RDT—Sens 46.3, Spec 98.2%

In this study, the use of Alere HS-RDT increased the sensitivity from 46% (conventional RDT) to 54%. The estimated cost of the Alere HS-RDT is around USD $2 in contrast to USD $1 for a conventional RDT. Is an 8% increase in sensitivity cost-effective, considering that the “highly sensitive” RDT missed nearly half of the *P. falciparum* infections detected by conventional qPCR?

**University of California**, San Francisco (UCSF) conducted a study in Champasak Province, Lao PDR bordering Cambodia in the south (unpublished data).

- HS-RDT vs. PCR *P. falciparum*
- Sens—69.8%, Spec—99.8%
- RDT vs. PCR *P. falciparum*
- Sens—68.3%, Spec—99.8%
- RDT vs. PCR *P. vivax*
- Sens—7.9%, Spec—98.8%

In this study, the performance of the HS-RDT and the conventional RDT were nearly identical, further raising questions regarding the benefit of the additional investment. Considering the high expectations placed in HS -RDT, the underwhelming performance, at least in the GMS, is thoroughly disheartening.

## Mass drug administration

Perhaps, historically the first public health intervention aimed at eliminating malaria has been the treatment of every single person in a target population [42, 43]. Such mass drug administrations have started with the introduction of effective anti-malarials at the beginning of the last century and will continue as long as effective anti-malarials are available irrespective of expert recommendations. The fundamental problem of the presumptive treatment of an entire population is the fact that only the infected fraction of the population directly benefits while the uninfected majority carry the burden of potential adverse effects of the treatment but without a direct benefit beyond the transient prophylaxis provided by the anti-malarial drugs. Such altruistic behaviour is often in conflict with human nature. Thus, establishing sufficient trust between the providers of the presumptive treatment and the target population is essential. Without the buy-in of the target population the coverage of mass drug administration can lead to disappointment (Box 3). There is only a single example of the elimination of malaria by mass drug administration, in an island population (n ≈ 1000) in Vanuatu, South Pacific [44]. Everywhere else malaria tended to resurge following a more or less pronounced nadir in transmission.

To assess the impact of targeted malaria elimination project named “Targeted Malaria Elimination” (TME) including a cluster randomised trial with dihydroartemisinin-piperaquine (DHA/piperaquine) MDA was conducted between 2013 and 2017 in 16 rural communities in Cambodia, Myanmar, Vietnam, and the Lao People’s Democratic Republic, where artemisinin resistance is prevalent. The results were presented during the meeting [45]. It is assumed that some mosquitoes, which ingested infected blood in the days prior to the MDA will survive until after the anti-malarial drug blood level is no longer high enough to clear a fresh *P. falciparum* inoculum. Perhaps more importantly, not all residents will be in the village on the same day, with some having travelled and others being too busy to participate. To address these limitations, three rounds of MDA 1 month apart were organized. Following intensive community engagement, establishing basic vector control measures and community-based case management, eight villages were randomized to receive early DHA/piperaquine MDA and eight villages were randomized as controls for 12 months, after which the control villages received deferred MDA. The MDA comprised three monthly rounds of three daily doses of DHA/piperaquine and, except in Cambodia, a single low dose of primaquine. *Plasmodium falciparum* prevalence was assessed at quarterly intervals by cross-sectional surveys of the entire population of each village using uPCR to detect *Plasmodium* infections. The MDAs were well tolerated, and individual protection was proportional to the number of completed MDA rounds. The primary outcome, *P. falciparum* prevalence by month 3 (M3), fell by 92% in early MDA villages and, over the same period, by 29% in control villages. Nevertheless, over the following 9 months, the *P. falciparum* prevalence increased to 3.3% (96/2881) in early MDA villages and to 6.1% (128/2101) in control villages. The main shortcoming of MDA is the resurgence following the initial decline in transmission. There are several factors that could be addressed to improve the impact of MDAs, but successful implementation at scale has considerable challenges.

1. *Achieving high coverage*: In the MDAs described here, women thought to be pregnant (first trimester in Myanmar, any trimester in other sites) and children under 6 months, approximately 2.5% and 3% of the target population were intentionally excluded from participation in MDA mostly due to regulatory safety concerns. Mobilizing the remaining population requires months of sensitization. A wide range of approaches to community engagement for MDAs has been trialled and evaluated. Health education, drama performance, and incentives (for individuals and communities) appear to have been successful, suggesting that the engagement modalities have to be adapted to the requirements of each community [46–52].

**Figure:**
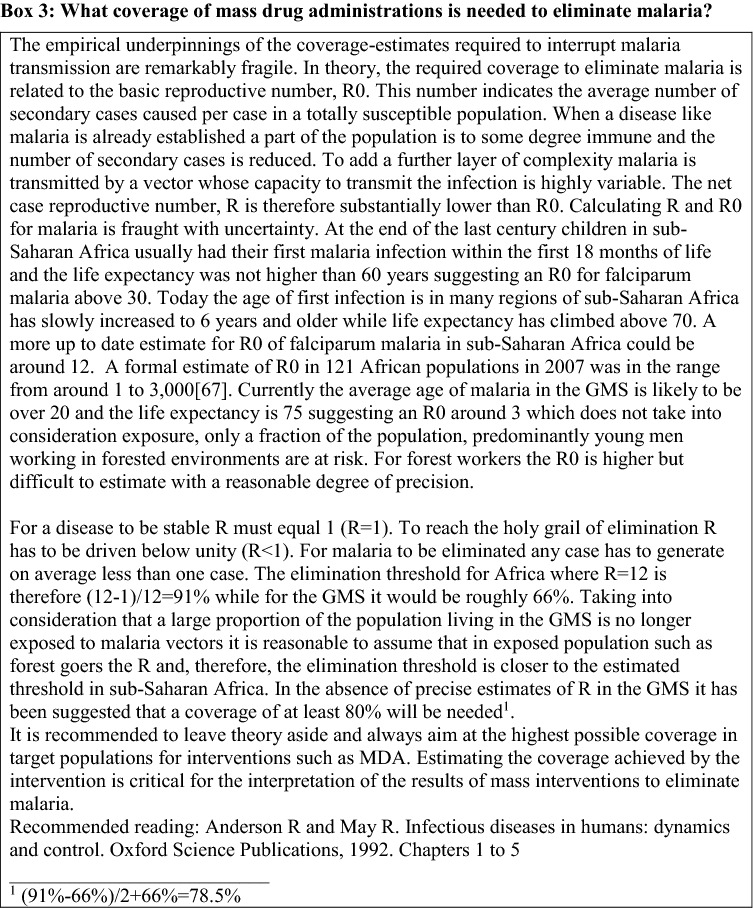
2. *The resources required*: the need for extended preparation and sensitization requires frequent skilled staff interaction with the communities. The three rounds of 3-day MDA require intense logistic planning to transport the essential manpower to the communities in a timely manner. Following MDA, skilled health care providers, equipped with the capacity to manage potential adverse events, must remain in participating communities. All these activities, in addition to the essential supplies, have funding implications.
3. *Which drugs to use*: Dihydroartemisinin/piperaquine with single low dose primaquine (0.25 mg/kg) used to be the most promising drug combination for MDA due to ease of administration, safety and tolerability, and quick parasite clearance rates. The MDAs described here were aimed at stopping the spread of anti-malarial resistance. Artemisinin resistance was first documented early this century in western Cambodia [53]. Resistance to the partner drug, piperaquine, was documented more recently and has since spread through parts of Cambodia, Laos and Vietnam. Whether DHA/piperaquine can still be used for MDA is controversial. Some researchers feel that the presence of resistance precludes further use, others take a more pragmatic approach suggesting that in the absence of a superior drug regimen the current effectiveness of DHA/piperaquine remains good enough (Box 4). Alternative drug regimens are under consideration but are unlikely to be as practical as DHA/piperaquine. The only other artemisinin-based combination suitable for MDA at this time could be pyronaridine/artesunate. The main challenge for a pyronaridine/artesunate regimen is remaining safety concerns. Liver toxicity due to pyronaridine/artesunate was suspected in earlier trials but these concerns have not been confirmed in subsequent trials [54, 55]. Nevertheless, comfortably rolling out this pyronaridine/artesunate in large, healthy populations would likely require further reassuring safety data. A future option for MDA regimens in the GMS could be triple artemisinin-based combinations therapy (TACT) [56]. Such combinations are currently in clinical trials, which appear positive, and could become an option if co-formulated. Safety and tolerability are a major concern in drug combination therapies and the potential for drug–drug interaction requires further, careful evaluation. Multidrug resistant *P. falciparum* has up to now only been reported and presents a problem for treatment in the eastern parts of the GMS (Southern Thailand, Cambodia, Vietnam, and Laos) [57, 58]. DHA/piperaquine remains a perfectly reasonable drug regimen for treatment or MDA outside of the areas affected by multidrug resistance.
4. *Prevention of the reimportation of infections*: besides the presumptive treatment of pre-existing falciparum infections, the anti-malarial drugs provide a window of prophylaxis. In an MDA regimen consisting of three rounds of three-dose DHA/piperaquine, this window of prophylaxis lasts approximately 3 months, after which the participants are once more susceptible to malaria. Two sources for new infections in MDA treated communities are, (a) residual infections in the community due to incomplete coverage and exclusion from MDA and, (b) the reimportation of infections by visitors and returning residents. In Cambodia, the TME trial tried to address this challenge by presumptively treating permanent visitors and returning residents with a single round of three-dose DHA/piperaquine [59]. Alternative approaches include the inclusion of the available, imperfect malaria vaccines [60]. Once malaria reaches elimination levels fewer and fewer villages harbour residual *P. falciparum* infections and the risk of re-importation becomes less and less relevant.
5. *Regulatory approval*: the potential lack of popularity of MDA in some target communities and concerns over safety have been noted by policy makers. For example, in Cambodia no approval could be obtained for the inclusion of single low dose primaquine in MDAs. In Myanmar regulatory approval was withdrawn for the continuation of MDAs in Karen state—which up to that point were part of a successful elimination strategy that had been implemented in 60 communities without adverse events and with good support from communities, evidenced by consistently high rates of participation.
6. *Which communities to target*: In the GMS there is a delicate balance between treating enough villages to eliminate the last remaining *P. falciparum* infections and overtreating villages where transmission has already been interrupted. Presumptive treatment of entire villages that did not report a single malaria case over years is unlikely to generate enthusiasm in the targeted communities. In the absence of empirical evidence and validated mathematical models an arbitrary threshold may be needed so that only communities with ongoing transmission are targeted. For example, in the above-mentioned malaria elimination study in Myanmar the threshold was defined as *“… the 90% CI upper limit of the sum of P. falciparum and P. vivax prevalence estimate was at least 40% and the corresponding value of the proportion of P. falciparum in the positive samples was at least 20%”* [21]. Other modalities to operate drug administration targeting highest risk groups instead of covering the entire population will miss significant portion of the infections. A simulation suggests that Risk-based Drug Administration (RDA) restricted to adult males (about 26% of the population) will only clear 15 of the 20 infections (Additional file 1: Figure S1).

Box 4: How effective remains DHA/piperaquine for MDA in the GMS? Three malaria research projects assessing the clearance rates of asymptomatic malaria were recently completed in Cambodia and the wider GMS by Médecins Sans Frontières (MSF), the Mahidol Oxford Research Unit (MORU), and the United States Armed Forces Institute for Medical Sciences (USAFRIMS). These studies give a rough snapshot of the current effectiveness of DHA/piperaquine against asymptomatic *Plasmodium falciparum* infections.

**MSF 2014** [62]—Chey Saen district, Preah Vihear province, northern Cambodia.

Of 16 *P. falciparum* samples collected from asymptomatic individuals, 11 contained sufficient *k13* gene for amplification by PCR and subsequent sequencing. 6/11 (55%) samples contained the mutant-type allele (C580Y). Piperaquine resistance was not explored. All positive cases were treated with a 3-day course of DHA/piperaquine and primaquine for G6PD wild type patients, parasitaemia at 28 days post-treatment was negative for all individuals.

**MORU 2014 to 2017** [45]—Battambang province, western Cambodia and Binh Phuoc, Vietnam.

**Table:** 

	All *P. falciparum*^b^	*P. falciparum*-*Pailin*^a^ only
Detected at base line	269	10
Completed at least one round of MDA	258	10
Follow-up specimen obtained	221	10
Remained positive after MDA	14	1
Clearance rates	94%	90%

^a^*Pfkelch* C580Y mutation and *Pfplasmepsin*2 amplification

^b^*P. falciparum* detection was undertaken using uPCR (ref)

**USAFRIMS 2018 (unpublished data)**

- Baseline—45 asymptomatic individuals *P. falciparum* PCR-positive
- Follow up (1 month)—18/45 (40%) remained *P. falciparum* positive (treatment with DP)
- Approximately 50% of all samples demonstrated *Pfplasmepsin2/3* amplification and 100% contained the *Pfkelch13* mutation.
- Directly observed treatment was carried out with all participants

The few, available data suggest a 100% clearance rate in the MSF studies in Cambodia, 90% clearance rate in MORU studies in Cambodia and Vietnam (no *P. falciparum* Pailin was detected in Myanmar and Laos) and 60% clearance rate in the USAFRIMS study in Cambodia. The studies use highly heterogenous methodologies. The MORU study used three rounds of DHA/piperaquine while the USAFRIMS and MSF used a single round (3 doses DHA/piperaquine).

The USAFRIMS study was conducted most recently suggesting that DHA/piperaquine has lost some of its effectiveness in the treatment of asymptomatic, low density *P. falciparum* infections in parts of the GMS. Nevertheless, following three rounds of MDA, the effectiveness may well be still 90% however the USAFRIMS data suggest that the residual DHA/piperaquine effectiveness in the GMS could be compromised and may be less than 90%. There is a need for a definitive trial to establish the current effectiveness of DHA/piperaquine against asymptomatic, low density *P. falciparum* Pailin infections in the GMS. In the absence of conclusive evidence that the MDA regimen can clear most *P. falciparum* infections, there may be reluctance to invest the massive resources required for MDAs in the GMS.

## Conclusions and future directions

The mundane but most promising approach to eliminate falciparum malaria remains the universal rapid access to early, accurate diagnosis and effective treatment of malaria episodes. Expanding and maintaining universal access to diagnosis and treatment remains the key to malaria control and ultimately malaria elimination in the GMS for the foreseeable future. Given the current spread of multidrug resistance and the subsequent failure of a number of artemisinin-based combinations, continuing this approach may ultimately depend on the introduction of new classes and combinations of anti-malarials.

MDA holds some promise for a rapid reduction of *P. falciparum* infections, but requires a considerable investment of resources and time to mobilize the target communities. Currently, the most practical drug regimen for MDA, three rounds of DHA/piperaquine, has lost some of its effectiveness against multidrug-resistant *P. falciparum* in Cambodia and Southern Vietnam. Mass screening and treatment benefits asymptomatic *P. falciparum* carriers by clearing chronic infections [63] but holds little promise for malaria elimination in theory or in practice. Hopes that “highly sensitive” diagnostic tests would provide substantial advances in screening programmes have shown to be misplaced. Further research is warranted to explore and optimize MDA using novel approaches to engage target communities and explore the addition of ivermectin and malaria vaccines [60, 64].

To reduce the burden of *P. falciparum* (and *Plasmodium vivax*) infections in people working in forested areas, novel approaches using malaria prophylaxis in forest goers needs to be explored. Forest work is one of the strongest risk factors for becoming infected with falciparum malaria in the GMS. Despite residual transmission in villages along the forest fringes, as witnessed by infected, young children who never visited the forest, it stands to reason that by eliminating sylvatic malaria, overall malaria elimination could be accelerated. Several approaches to protect forest workers are currently under consideration. These include the early diagnosis and effective treatment of forest workers through peer health care providers traveling with the workers. Another strategy is the provision of anti-malarial prophylaxis for those workers spending extended periods in the forest [11]. Of critical importance for such a strategy would be an effective drug regimen. The recently licensed 8-aminoquinoline, tafenoquine could play a key role in such a prophylaxis programme as it would not only protect against *P. falciparum* but also clear *P. vivax* hypnozoites. Like primaquine, the other licensed 8-aminoquinoline, tafenoquine carries a risk of triggering haemolysis in G6PD-deficient individuals. The prevalence of G6PD deficiency is highly heterogeneous, between 2 and 16% of residents in the GMS may have sufficiently severe G6PD deficiency to exclude them from any exposure to 8-aminoquinolines [65–67]. To use tafenoquine safely as a prophylaxis in the GMS it would be essential to have access to robust diagnostic tools which identify G6PD deficient individuals. Such diagnostic tools are forthcoming and once policymakers are sufficiently comfortable with the safety and effectiveness of tafenoquine it may play an increasing role in malaria elimination in the GMS.

It may be worthwhile to mention why *P. vivax* infections were not discussed in the meeting. The lifecycle of *P. vivax* differs from *P. falciparum* in that it includes a hypnozoite stage that transform vivax malaria into a recrudescent chronic illness which requires a radical cure with 8-aminoquinolines (e.g. primaquine or the above mentioned tafenoquine) to eliminate this disease. Accordingly, public health strategies to eliminate vivax malaria have to differ from falciparum elimination strategies. But there should be no doubt that the elimination of vivax malaria is an urgent necessity and should be integrated with falciparum malaria elimination to achieve the elimination all malarias from the GMS, southeast Asia, and all other co-endemic regions.

In summary, by reviewing recent evidence, several important conclusions on accelerating malaria elimination in the GMS were reached and potential future directions were discussed. There is a hopeful atmosphere that widespread access to early accurate diagnosis and the effective treatment of malaria episodes can and should be continued and intensified around the high-risk forested areas along with the ongoing roll-out of mobile malaria posts, while new strategies are being explored.

## Supplementary information

**Additional file 1: Figure S1.** Number of *P. falciparum* infections treated using variations of mass drug administrations and screen and treat strategies

## Data Availability

Not applicable for this meeting report.

## Abbreviations

ACT: Artemisinin-based combination therapy
CNMCP: Cambodian National Malaria Control Programme
DHA: Dihydroartemisinin
FG: Forest goer
FSAT: Focal screening and treatment
GMS: Greater Mekong Subregion
HSRDT: “Highly sensitive” rapid diagnostic tests
IPC: Institut Pasteur of Cambodia
IPTfg: Intermittent Presumptive Treatment of forest goers
LSHTM: London School of Hygiene and Tropical Medicine
MC: Malaria Consortium
MDA: Mass Drug Administration
MSAT: Mass screening and treatment
MSF: Médecins Sans Frontières
OD: Operational district
SSA: Sub-Saharan Africa
UCSF: University of San Francisco
uPCR: Ultrasensitive PCR
URC: University Research Company
