# Supplementary material for: Tools to accelerate falciparum malaria elimination in Cambodia: a meeting report
Source: Malar J. 2020 Apr 15;19:151. doi: 10.1186/s12936-020-03197-6 (PMC7161105; doi:10.1186/s12936-020-03197-6)

Figure S1: Number of P. falciparum infections treated using variations of mass drug administrations and screen and treat strategies.

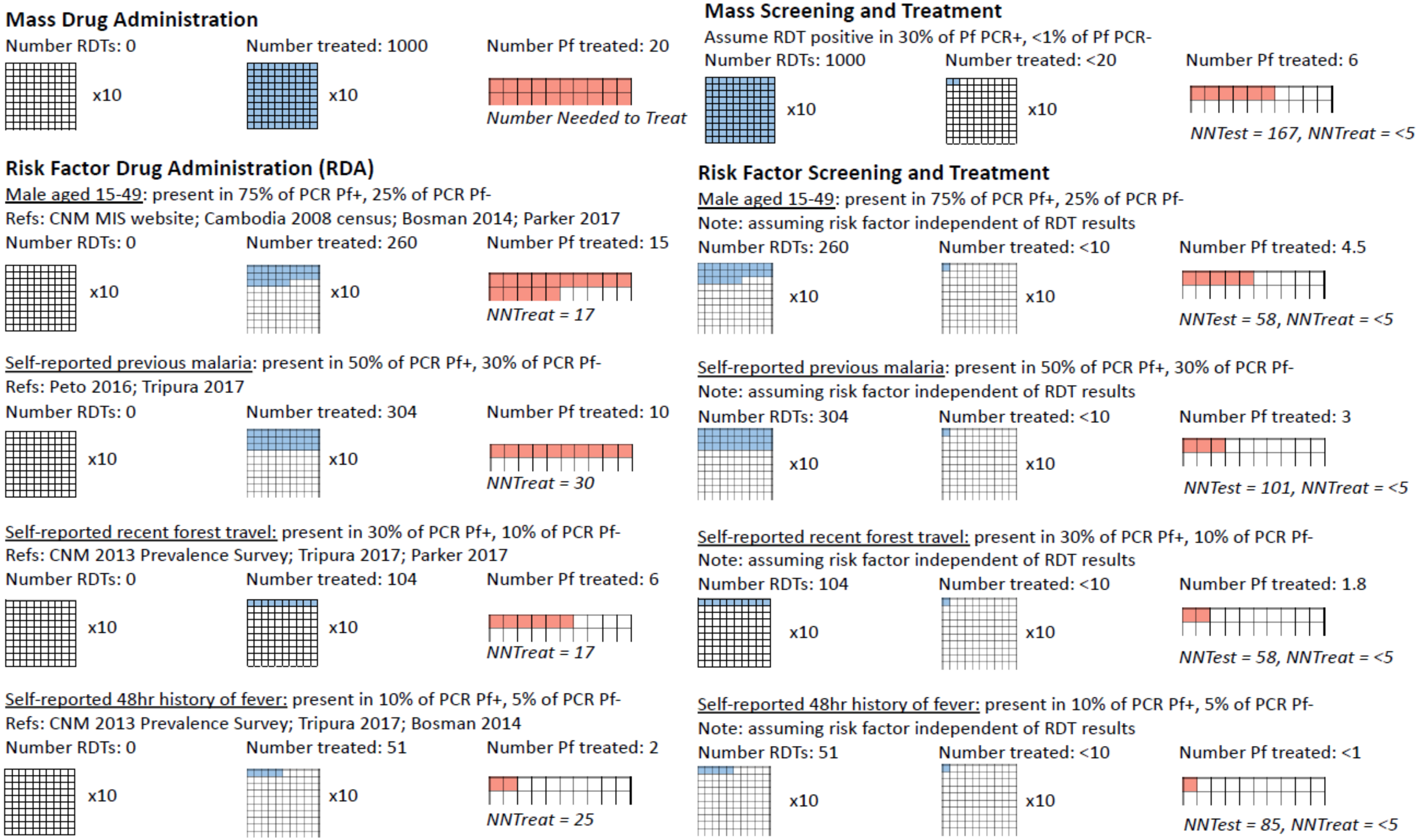

Supplement: Supplementary file 1 — Additional file 1: Figure S1. Number of P. falciparum infections treated using variations of mass drug administrations and screen and treat strategies [file 12936_2020_3197_MOESM1_ESM.pdf]
